# Validity and Reliability of Health Belief Model Questionnaire for Promoting Breast Self-Examination and Screening Mammogram for Early Cancer Detection

**DOI:** 10.31557/APJCP.2019.20.9.2865

**Published:** 2019

**Authors:** Norfariha Che Mohamed, Soo-Foon Moey, Bee-Chiu Lim

**Affiliations:** 1 *Department of Diagnostic Imaging and Radiotherapy, Kulliyyah of Allied Health Sciences, International Islamic University Malaysia (IIUM), Kuantan Campus,*; 2 *Clinical Research Centre, Hospital Tengku Ampuan Afzan (HTAA), Kuantan, Pahang, Malaysia.*

**Keywords:** Health belief model, validation, reliability, breast self-examination, mammogram, breast cancer

## Abstract

**Background:**

Early detection of breast cancer is essential in improving overall women’s health. The researchers sought to develop a comprehensive measure that combined the basic components of the health belief model (HBM) with a focus on breast self-examination (BSE) and screening mammogram amongst women.

**Methods:**

Questionnaire items were developed following a review of relevant literature of HBM on BSE and screening mammogram. The sampling frame for the study was Malaysian women aged 35 to 70 years old, living in Kuantan, Pahang and able to read or write in Bahasa Malaysia or English. As such, 103 women were randomly selected to participate in the study. Tests of validity using exploratory factor analysis (EFA) and reliability were subsequently performed to determine the psychometric properties of the questionnaire.

**Results:**

The EFA revealed nine factors (self-efficacy of mammogram, perceived barriers of BSE and mammogram, perceived susceptibility of breast cancer, perceived severity of breast cancer, cues to action for mammogram screening, perceived benefits of BSE, health motivation, perceived benefits of mammogram and self-efficacy of BSE) containing 54 items that jointly accounted for 74.2% of the observed variance. All nine factors have good internal consistency with Cronbach’s alpha ≥ 0.8. Fifty-four items remained in the final questionnaire after deleting 13 problematic items. The scale also showed good convergent and discriminant validity.

**Conclusion:**

The findings showed that the designed questionnaire was a valid and reliable instrument for the study involving women in Kuantan, Pahang. The instrument can help to assess women’s beliefs on BSE adoption and mammogram screening in health care practice and research.

## Introduction

Breast cancer is the commonest cancer amongst women globally with increased incidence in recent years. In 2018, it is estimated that there will be about 2.1 million new cases of breast cancer throughout the world which makes up for almost one in four cancer cases among women (Bray et al., 2018). In Malaysia, the National Cancer Registry reported the Age Standardized Rate (ASR) of female breast cancer was 31.1 per 100,000 population from 2007 to 2011, which is highest between age group of 50 to 59. Malaysia being a multiracial country, variations in ASR do exist with the ASR amongst the Chinese being the highest at 41.5 per 100,000 population. Additionally, the ASR for the Indians was 37.1 and the Malays was the lowest at 27.2 per 100,000 population (Ministry of Health Malaysia, 2016). As such, the ASR of breast cancer has brought about high concern amongst health professionals to detect breast cancer at an early stage. 

Increase effort to detect early breast cancer is crucial to improve women’s health and to decrease the cost related to cancer death (Noroozi et al., 2011), whereby early detection itself can consequently lead to an increase in the survival rate (Yarbrough and Braden, 2001; Kulakci et al., 2015; Mohamed et al., 2016). Breast cancer screening facilitates early detection which includes age appropriate mammography, clinical breast examination (CBE) and breast self-examination (BSE) which currently are the only health protection alternatives available (Yarbrough and Braden, 2001). Monthly BSE is an important screening activity for early diagnosis of breast cancer (George, 2000; Shahroodi et al., 2015) and it has been reported in the literature that 90% of all breast cancers have been detected by the patients themselves as they know the usual structure of their breasts (Yucel et al., 2014). 

As the most effective and standardized screening method, mammography is necessary for achieving the highest level of success in breast cancer screening (Mirzaei-Alavijeh et al., 2018). Findings from previous studies indicated that older women undertake mammography screening much more than younger ones (Yusof et al., 2014; Mirzaei-Alavijeh et al., 2018). This discrepancy should never exist as a baseline mammogram should be obtained by age 40, with yearly mammogram after the age of 40 (George, 2000). The Malaysian Clinical Practice Guidelines on the Management of Breast Cancer (2010) recommends that screening women which are at a high risk for breast cancer should be done from the age of 30 years utilizing both magnetic resonance imaging (MRI) and mammography as they are more effective than mammography alone. However, in low and intermediate risk women aged 40 to 49 years old breast cancer screening using mammography is highly recommended (Ministry of Health Malaysia, 2010; Mahmud and Aljunid, 2018). Nevertheless, in places that lack screening mammography, CBE is one of the most important methods for breast cancer detection where it identifies some cancers not found by mammography (Farid et al., 2014).

Adoption of breast screening practices is linked to perceptions of risk, benefit, and barriers through a reasoning process that includes personal and social influences and attitudes (Yarbrough and Braden, 2001). Since the early 70’s, many theories have been proposed to help explain health-related behaviors. The health belief model (HBM) with its own core constructs is one of the most prominent which conceptualized potential barriers or facilitators for a desired health adoption behavior. Over the years, HBM constructs have been used widely to measure breast cancer screening adoption practices in mammography mainly through translations and adaptations of the Champion (1999) Health Belief Model Scale (Stewart et al., 2009). This scale has been tested for the psychometric properties, mostly in western and other cultures (Secginli and Nahcivan, 2004).

However, a study to check on the validity and reliability of the Champion’s Health Belief Model Scale (CHBMS) for breast cancer screening was carried out in Malaysia. The study concluded that CHBMS is a valid and reliable instrument in measuring health beliefs pertaining to breast cancer and its’ screening methods (Parsa et al., 2008). A subsequent study conducted on Malaysian student population further acknowledged that CHBMS is a suitable instrument in evaluating the beliefs of breast cancer and breast cancer screening among women, especially young female (Akhtari-zavare et al., 2018). According to CHBMS, to participate in breast cancer screening a woman must perceive to be susceptible to various perceptions that can influence attitudes and practices, benefits and barriers (Parsa et al., 2008). 

An individual’s effort to improve health is often expressed by the individual health seeking behavior with the use of preventive practices. Hence, CHBMS suggests that women with enough knowledge about preventability of breast cancer are more likely to seek preventive care by adopting breast screening practices such as mammography (Chukmaitov et al., 2008). In Malaysia, although awareness of breast cancer is high, this did not correspond well with awareness of available screening measures (Yusof et al., 2014). According to studies conducted in the urban and sub-urban localities of Terengganu, Selangor and Kuala Lumpur, mammography screening uptake was between 10.5% and 31.9% amongst the population, while amongst women in rural localities of Perak and Pahang the mammography screening uptake was found to be less than 10% (Mahmud and Aljunid, 2018).

An individual tends to adopt healthier behaviors when they perceive the new behavior will reduce their chance of acquiring a disease (Mohamed et al., 2016). However, many factors have been reported as contributing to the delay in adopting healthier behavior and to seek medical care which includes religiosity, spirituality and fatalistic beliefs (Gullatte et al., 2010; Shah et al., 2017). An individual with higher levels of religiosity tends to perceive his or her disease less seriously as they believe what occurred in their lives is attributable to God, their protector. This is in line with findings from a study, which indicated that women that practice frequent religious activities such as reading religious materials had significantly poorer knowledge of breast cancer. Additionally, fatalistic beliefs of women that breast cancer was ‘fated’ and unavoidable may also result in their refusal to seek any knowledge or treatment about their illness (Shah et al., 2017). Fatalistic beliefs about cancer prevention can be a significant barrier to an individual’s likelihood of engaging in cancer prevention behaviors. A study showed that rural residents were more likely to endorse multiple fatalistic beliefs about cancer prevention and as such are less likely to adopt cancer screening compared to the urban residents (Befort et al., 2013).

With regards to predicting BSE and mammography screening practices amongst women, this paper describes the assessment of validity and reliability of the HBM questionnaire used in developing a model to promote women’s behavior towards BSE and screening mammogram in Kuantan, Pahang. It is vital to develop an instrument that is culturally appropriate for all women in Malaysia. Thus, the objective of the study is to evaluate the validity and reliability of the subscales of HBM. Findings from this study may provide some information that enables the healthcare team to better understand women’s beliefs related to breast cancer screening behavior and potentially encourage them to increase screening practices by adopting BSE and mammography. 

## Materials and Methods

*Item generation*

A self-administered questionnaire was developed, keeping in mind the subscales of HBM to explore cancer control behavior amongst women. The initial item pool for the HBM questionnaire to promote BSE and screening mammogram was generated using a comprehensive review of existing instruments and health belief theory (Champion, 1984; Fulton et al., 1991; Marmara et al., 2017). 

To establish content validity, the questionnaire was administered to a panel of five health professional experts which includes two professors, one radiologist specializing in diagnosis and screening of breast cancer, an English lecturer and a research scholar in women’s health. The members were selected based on their experiences and were requested to review the 67 items for clarity and in ensuring the items fit the subscales and definition. The questionnaire was originally in English language and was translated into Malay using a back-translation technique executed by the experts. To do this, one expert took the original version of the questionnaire and translated all the items into Malay. Another expert then translated the Malay version into English. The back-translated version was compared to the original version of the scale to check for the similarity of meaning and grammar (Secginli and Nahcivan, 2004; Parsa et al., 2008). The evaluation of item wording, response format and instrument length were carried out by the expert panels to ensure that all items were deemed to be relevant and appropriate.

**Table 1:** Demographic Characteristics of the Participants (n = 103).

Characteristics	Frequency	%
Age (years)		
< 40	39	37.8
40-49	49	47.6
> 50	15	14.6
Ethnic		
Malay	92	89.3
Non-Malay	11	10.7
Religion		
Muslim	93	90.3
Non-muslim	10	9.7
Marital status		
Single	26	25.2
Married	71	69.0
Others	6	5.8
Education level		
No formal education	0	0.0
Primary education	1	1.0
Secondary education	21	20.4
Tertiary education	81	78.6
Occupation		
Government	61	59.2
Private employee	25	24.3
Self-employed	7	6.8
Home maker	10	9.7
Family income (RM)		
< 1,000	5	4.9
1,000-2,999	28	27.2
3,000-5,999	47	45.6
6,000-9,999	14	13.6

RM, Ringgit Malaysia

**Table 2:** Description on the Factors and Items, Communalities, Factor Loading, Corrected Item-Total Correlation and Cronbach’s Alpha (n = 103)

Factor and items	Communalities	Factor Loading	Corrected item-total correlation	Cronbach’s Alpha
F1 (Self-efficacy Mammogram)				0.958
R1 I can arrange transportation to get a mammogram/ *Saya boleh menguruskan pengangkutan untuk mendapatkan mammogram*	0.925	0.946	0.85	
R2 I can arrange other things in my life to have a mammogram/ *Saya boleh menguruskan hal-hal lain dalam hidup saya untuk melakukan mammogram*	0.931	0.9	0.834	
R3 I can talk to other people at the mammogram center about my concerns/ *Saya boleh bercakap dengan orang lain di pusat mammogram mengenai kebimbangan saya*	0.885	0.967	0.846	
R4 I can get a mammogram even if I am worried/ *Saya boleh mendapatkan mammogram walaupun saya bimbang*	0.922	0.878	0.854	
R5 I can get a mammogram even if I don’t know what to expect/ *Saya boleh mendapatkan mammogram walaupun saya tidak tahu apa yang bakal berlaku*	0.828	0.817	0.792	
R6 I can find a way to pay for a mammogram/ *Saya boleh mencari cara untuk membayar mammogram*	0.946	0.838	0.85	
R7 I can make an appointment for a mammogram/ *Saya boleh membuat temujanji untuk mammogram*	0.957	0.916	0.899	
R8 I know I can get a mammogram if I really want to/ *Saya tahu saya boleh mendapatkan mammogram jika saya benar-benar mahu*	0.925	0.709	0.799	
F2 (Barriers BSE and Mammogram)				0.989
M1 Performing BSE is time consuming/ *Menjalankan pemeriksaan sendiri payudara memakan masa yang lama*	0.786	0.665	0.623	
M3 No suitable place at home to perform BSE/ *Tiada tempat yang sesuai di rumah saya untuk melakukan pemeriksaan sendiri payudara*	0.891	0.744	0.695	
M4 Feeling of shame and embarrassment when performing BSE/ *Saya berasa malu ketika melakukan pemeriksaan sendiri payudara*	0.864	0.714	0.711	
M5 Performing BSE is tedious/ *Menjalankan pemeriksaan sendiri payudara adalah leceh*	0.855	0.798	0.802	
M6 Performing BSE increase my anxiety about having breast cancer/ *Menjalankan pemeriksaan sendiri payudara menaikkan kadar kebimbangan saya*	0.87	0.723	0.622	
M7 I think getting breast cancer is fated and BSE will not change it/ *Bagi saya menghidapi kanser payudara itu adalah takdir dan melakukan pemeriksaan sendiri payudara tidak akan mengubahnya*	0.808	0.578	0.474	
Q2 Having a routine mammogram would make me worry/ *Menjalani rutin mammogram rutin akan membuatkan saya risau*	0.82	0.588	0.593	
Q3 Having a mammogram would be embarrassing/ *Menjalani mammogram akan memalukan*	0.797	0.702	0.751	
Q4 Having a mammogram would take too much time/ *Menjalani pemeriksaan mammogram mengambil masa yang sangat lama*	0.816	0.763	0.728	
Q5 Having a mammogram would be painful/* Pemeriksaan mammogram menyakitkan*	0.723	0.618	0.585	
F3 (Susceptibility)				0.949
K1 I am susceptible to breast cancer in the future/ *Saya mungkin terdedah dengan kanser payudara di masa hadapan*	0.886	0.839	0.81	
K2 I feel that I am susceptible to breast cancer/ *Saya merasakan saya mudah terdedah kepada kanser payudara*	0.957	0.928	0.918	
K3 I think that I am susceptible to breast cancer more than anyone/* Saya merasakan saya mudah terdedah kepada kanser payudara berbanding orang lain*	0.925	0.919	0.882	
K4 My personal chance of getting breast cancer is high/ *Kemungkinan saya menghidapi kanser payudara adalah tinggi*	0.923	0.915	0.876	
K5 I am highly susceptible to breast cancer in next 10 years / *Saya sangat mudah terdedah kepada kanser payudara dalam masa 10 tahun akan datang*	0.858	0.801	0.826	
F4 (Severity)				0.908
I2 When I think about breast cancer my heart beat faster/ *Jantung saya berdegup laju apabila memikirkan tentang kanser payudara*	0.899	0.825	0.811	
I3 I am afraid even to think about breast cancer/ *Saya merasa takut memikirkan tentang kanser payudara*	0.901	0.859	0.847	
I4 All my life will be changed if I got breast cancer/ *Kehidupan saya akan berubah jika saya menghidapi kanser payudara*	0.827	0.749	0.671	
I5 The thought of breast cancer scares me/ *Apabila saya memikirkan tentang kanser payudara, ia menakutkan saya*	0.922	0.912	0.849	
F5 (Cues to action Mammogram)				0.907
P1 Effective for early detection of breast cancer/ *Berkesan untuk pengesanan awal kanser payudara*	0.817	0.736	0.685	
N4 I can discover breast tumor at size of small pea/ *Saya boleh mengenal pasti saiz ketumbuhan sebesar kacang pea pada payudara saya*	0.882	0.747	0.73	
N5 I am able to differentiate between normal and abnormal breast tissue through BSE/ *Saya mampu untuk membezakan antara tisu payudara normal dan tidak normal dengan menjalankan pemeriksaan sendiri payudara*	0.75	0.669	0.587	
P2 Can detect lumps that doctor can’t find/ *Mampu untuk mengesan bonjolan yang tidak dijumpai oleh doktor*	0.79	0.647	0.685	
S1 Reminder letters would help me to get a mammogram/ *Surat peringatan akan membantu saya mendapatkan mammogram*	0.956	0.728	0.821	
S2 Reminder phone calls or text messages would help me to get a mammogram/ *Peringatan panggilan telefon atau mesej teks akan membantu saya mendapatkan mammogram*	0.965	0.666	0.85	
S3 Routine educational talks regarding breast cancer awareness would help me to get a mammogram/ *Perbincangan pendidikan rutin mengenai kesedaran kanser payudara akan membantu saya mendapatkan mammogram*	0.975	0.775	0.905	
S4 I feel confident that if I had a mammogram done, any abnormalities in my breasts will be detected/* Saya berasa yakin bahawa jika saya melakukan mammogram, sebarang kelainan pada payudara saya akan dikesan*	0.971	0.673	0.886	
S5 I can arrange other things in my life to get a mammogram/ *Saya boleh menguruskan perkara lain dalam kehidupan saya untuk mendapatkan mammogram*	0.895	0.596	0.763	
F6 (Benefits BSE)				0.89
L1 Performing BSE monthly help in early detection of breast cancer/ *Menjalankan pemeriksaan sendiri payudara secara kadar bulanan dapat membantu dalam pengesanan awal kanser payudara*	0.93	0.731	0.723	
L2 Performing BSE monthly help in detection of tumors before going to the doctors/ *Menjalankan pemeriksaan sendiri payudara secara kadar bulanan dapat membantu dalam pengesanan awal kanser payudara sebelum berjumpa doktor*	0.943	0.738	0.778	
L3 Performing BSE monthly will decrease complications of breast cancer if I got breast cancer/ *Menjalankan pemeriksaan sendiri payudara secara kadar bulanan boleh mengurangkan komplikasi terhadap kanser payudara sekiranya saya menghidapinya*	0.888	0.796	0.786	
L4 Performing BSE decrease the chance of surgery if I got breast cancer/ *Menjalankan pemeriksaan sendiri payudara dapat mengurangkan kemungkinan pembedahan sekiranya saya menghidap kanser payudara*	0.893	0.717	0.64	
L5 Performing BSE decrease the anxiety about breast cancer/ *Menjalankan pemeriksaan sendiri payudara dapat mengurangkan keresahan tentang kanser payudara*	0.87	0.711	0.771	
F7 (Motivation)				0.829
J2 I wish to discover health problem that occur early/ *Saya ingin mengetahui masalah kesihatan saya dengan lebih awal*	0.768	0.568	0.533	
J3 I always seek new information that improves my health/ *Saya selalu mencari informasi baru untuk meningkatkan kesihatan saya*	0.908	0.896	0.788	
J4 I feel the importance of activities that improve my health/ *Saya percaya kepentingan aktiviti untuk meningkatkan kesihatan saya*	0.892	0.823	0.712	
J6 I exercise at least 3 times a week/ *Saya beriadah sekurang-kurangnya 3 kali seminggu*	0.787	0.708	0.664	
J7 I perform annual medical check-up/ *Saya melakukan pemeriksaan perubatan setiap tahun*	0.855	0.734	0.649	
F8 (Benefits Mammogram)				0.909
P4 When I get a mammogram, I do not worry as much about breast cancer/ *Apabila saya menjalani mammogram, saya tidak terlalu risau tentang kanser payudara*	0.902	0.695	0.756	
P5 Having a mammogram will help me find lumps early in my breasts/ *Menjalani mammogram mampu membantu saya dalam mengesan benjolan dengan lebih awal di payudara saya*	0.908	0.552	0.741	
P6 If I find a lump through a mammogram, the treatment for breast cancer may not be as bad/ *Sekiranya saya menjumpai benjolan melalui mammogram, rawatan untuk kanser payudara itu mungkin tidak seteruk*	0.88	0.686	0.791	
P7 Having a mammogram will decrease my chances of dying from breast cancer/ *Menjalani pemeriksaan mammogram akan mengurangkan risiko kematian akibat kanser payudara*	0.914	0.824	0.768	
P8 Having a mammogram will help me find a lump before it can be felt by myself or a health professional/ *Menjalani pemeriksaan mammogram akan membantu saya menjumpai benjolan sebelum ia boleh dirasa oleh saya atau pakar kesihatan*	0.915	0.859	0.821	
F9 (Self-efficacy BSE)				0.878
N1 I am confident in performing BSE correctly/ *Saya yakin dapat melakukan pemeriksaan sendiri payudara dengan betul*	0.881	0.662	0.654	
N2 I can use the correct part of my fingers when performing BSE/* Saya tahu menggunakan jari yang betul dalam melakukan pemeriksaan sendiri payudara*	0.911	0.793	0.763	
N3 I am confident I can discover breast tumours by performing BSE/ *Saya yakin saya boleh menemui ketumbuhan di payudara dengan melakukan pemeriksaan sendiri payudara*	0.883	0.901	0.829	

F, factor; I, J, K, L, M, N, P, Q, R; S, items under the nine distinct factors

**Table 3:** Correlation between Factors (n = 103)

Factor	1	2	3	4	5	6	7	8	9
1	1								
2	-0.250*	1							
3	0.067	0.159	1						
4	0.114	-0.022	0.061	1					
5	0.607***	-0.237*	0.092	0.079	1				
6	0.192	-0.128	-0.012	0.133	0.368***	1			
7	0.187	-0.226*	-0.033	-0.069	0.297**	0.332***	1		
8	0.340***	-0.037	-0.027	0.082	0.415***	0.441***	0.359***	1	
9	0.315**	-0.149	0.089	0.081	0.281**	0.225*	0.279**	0.183	1

*, p < 0.05; **, p < 0.01; ***, p < 0.001

The 67 items for this study representing 14 factors were scored on a Likert scale ranging from 1 (strongly disagree) to 10 (strongly agree). Refer to Appendix 1. All the items in the subscales are positively worded except for items under the subscales of perceived barriers of BSE and mammogram. 

*Participants*

The sampling frame for the study was Malaysian women aged 35 to 70 years old, living in Kuantan, Pahang and able to read or write in Bahasa Malaysia or English. As such, a total of 103 women were selected by means of simple random sampling to participate in the study. As this is an exploratory study to determine the factor structures and reliability of the scale, the sample size (n=103) was approximately 100% more than the 10% of the sample size of 520 participants planned for the actual study as suggested by Connelly (2008).

The consented participants were provided with the generated questionnaire and they were given time to fill their responses at their will in a private and confidential setting. The questionnaire took approximately 10 to 15 minutes to be completed. Univariate normality check was done on each item, visually by inspection of histogram with normality curve and box-and-whisker plot.

*Exploratory factor analysis*

After the questionnaires were anonymously returned to the researcher, the data was compiled and checked for internal structure evidence of validity using the Statistical Package for Social Science (SPSS, version 21). Exploratory factor analysis (EFA) was carried out to explore the construct validity of HBM subscales. Principal axis factoring (PAF) method was utilised to identify subscales within the item pools and to exclude items that did not conceptually group in the subscales. Bartlett’s Test of Sphericity (p < 0.05) and the Kaiser–Meyer–Olkin (KMO) statistics with the recommended value of 0.6 was applied to measure sampling adequacy and appropriateness of the factors extracted (Tabachnick and Fidell, 2007; Hair Jr et al., 2009). Factors with eigenvalues above one were extracted and an oblique Promax rotation was used to determine correlation between factors (Norman and Streiner, 2000). 

Items with loading factor greater than 0.4 were considered as an acceptable loading factor under a component. Factor loading was used to identify items with low correlation to its individual rotated factor for further re-specification (Yong and Pearce, 2013). Factor pattern matrix were interpreted similarly to factor loadings (Hair Jr et al., 2009). These coefficients are partial standardized regression coefficients of each item with a factor. 

*Convergent and discriminant validity*

Convergent and discriminant validity were used in determining construct validity. Internal consistency of the HBM subscales was assessed using Cronbach’s alpha coefficient to provide evidence of convergent validity between the items in the subscales (Trochim, 2006). The item-total correlation was also used as a method for checking the homogeneity of the scale. An alpha coefficient > 0.7 was considered acceptable for internal consistency reliability whilst an item should correlate with the total score above 0.3 (Streiner and Norman, 2008). However, discriminant validity was assessed among factors extracted using factor correlations. The correlation among factors ≤ 0.85 to establish discriminant validity between constructs was suggested (Kline, 2011).

## Results

*Participant demographics*

The mean age of the 103 participants was 42.4 years (SD = 6.7), range 35 to 70 years with the majority of the participants between 35 and 40 years. Most women were Malay (89.3%), Muslim (90.3%) and were married (69%). More than half of the women had tertiary education (78.6%). Additionally, most of the women worked in the government sector (59.2%). Approximately 46% of the participants reported a monthly family income between RM 3,000 to RM 5,999. Details of the demographics are shown in Table 1.

*Exploratory factor analysis*

All the items were found not normally distributed when using the univariate normality check. Thus, EFA method using PAF was used for factor extraction. The initial KMO measured was 0.626, and the Bartlett’s Test of Sphericity was significant (χ^2^= 7444.322, p < 0.001), indicating adequacy of sample for EFA. Initially, the 67-item scale and 14 factors which comprised 79.4% variance showed eigenvalues above 1.0. The communalities (extraction) showed that it was more than 0.50 for all items which further confirmed that each item shared some common variance with other items. Therefore, factor analysis was conducted for all 67 items.

The initial 14-factor solution was explored repeatedly by assessing the item performance and factor loadings. Elimination of the problematic items was done in a step-by-step process. During several steps of EFA, a number of factors were fixed and 13 items were eliminated one by one because these items did not contribute to a simple factor structure and failed to meet the minimum criteria of having factor loading ≥ 0.40 or cross loading on other factors. The eliminated items were I1 (“*I think I will not live more than 5 years with breast cancer*”), J1 (“*Keeping good health is important to me*”), J5 (“*I consume a balanced diet*”), M2 (“*Performing BSE is unpleasant to me*”), O1 (“*I have heard about BSE from the mass* *media: books/ magazines/ pamphlets/ health education materials/ television or video*”), O2 (“*I have heard about BSE from talking with: friends/ partner/ doctor/ healthcare provider*”), O3 (“*Having known someone who had breast cancer made me do BSE*”), O4 (“*Having a close relative who had breast cancer made me do BSE*”), P3 (“*More effective than clinician or breast self-examination*”), Q1 (“*Having a routine mammogram would make me anxious about breast cancer*”), Q6 (“*Having a mammogram would cost too much*”), Q7 (“*Screening mammogram program is difficult to fit into my schedule*”) and S6 (“*In case I need a mammogram, I will find a place to get it done*”). A final factor solution that consisted of a 54-item scale loading on nine distinct constructs was obtained. These constructs jointly accounted for 74.2% of the observed variance. 

A summary of the nine-factor solution is shown in Table 2. The items under the nine distinct constructs were further reviewed and the factors labelled. Factor 1 consisted of 8 items (R1-R8) and named “*self-efficacy of mammogram*”, factor 2 consisted of 10 items (M1, M3-M7; Q2-Q5) and labelled as “*perceived barriers of BSE and mammogram*”, factor 3 consisted of 5 items (K1-K5) and was named “*perceived susceptibility of breast cancer*”. Factor 4 is the “*perceived severity of breast cancer*” which have 4 items (I2-I5), while factor 5 contained 7 items (P1-P2; S1-S5) and named “*cues to action for mammogram screening*”. 

Factor 6 consisted of 5 items (L1-L5) and named “*perceived benefits of BSE*”. Factor 7 contained 5 items (J2-J4; J6-J7) and named “*health motivation*”, factor 8 is the “*perceived benefits of mammogram*” which consisted of 5 items (P4-P8) and lastly, factor 9 is the “*self-efficacy of BSE*” which included 5 items (N1-N5). The factor analysis of the revised 54-item of HBM scale had a KMO measure of sampling adequacy of 0.690 and Bartlett’s Test of Sphericity (χ^2^= 5704.003, p < 0.001) with the KMO showing a slight improvement from the initial 14-factor solution. 

*Convergent and discriminant validity*

After EFA, the reliability coefficient for each scale was calculated using Cronbach’s alpha coefficients. In this study, all items met these criteria with the alpha coefficients of the scales ranging from 0.829 to 0.989 indicating good reliability with item-total correlation greater than 0.30 while factor loadings in each factor were more than 0.40 (refer to Table 2). As such, the items retained showed convergent validity towards its subscales. Thus, no items of the instrument were omitted in this phase. The discriminant validity was shown in Table 3. The correlations among the nine factors ranged from -0.022 to 0.607. All factor correlations are less than 0.85. Hence, there is no multi-collinearity (factors are distinct from each other) between the items.

## Discussion

In this study, the HBM scales have been developed and evaluated for use in BSE and screening mammogram among women in Kuantan, Pahang. Nine factors related to HBM were identified from the EFA. The factors were self-efficacy of mammogram, perceived barriers of BSE and mammogram, perceived susceptibility of breast cancer, perceived severity of breast cancer, cues to action for mammogram screening, perceived benefits of BSE, health motivation, perceived benefits of mammogram and self-efficacy of BSE were derived in the new scale as the main components of HBM. The final version of the scale consisted of 54 items. The number of items in the validated questionnaire is close to the 57 items utilized in the study carried out by Taymoori and Berry (2009) to validate CHBMS for breast cancer screening behaviors among Iranian women. Further, studies conducted by Parsa et al., (2008) and Akhtari-zavare et al., (2018) on validation studies on CHBMS employed 63 and 67 items respectively. However, a study conducted by Mikail and Petro-Nustas (2001) to validate the questionnaire on transcultural adaptation of CHBMS amongst Jordanian women only employed 43 items. Additionally, only 36 items had been utilized by Lee et al., (2002) to validate CHBMS in Korean. Given this, the number of items for the instrument in the current study can be considered as good enough but maybe refined to make it less troublesome and more manageable for the participants.

The EFA indicated that the model explained 74.2% of the observed variances which is well above previous studies that assessed the validity using the model from CHBMS (Mikail and Petro-Nustas, 2001; Lee et al., 2002; Secginli and Nahcivan, 2004; Zelviene and Bogusevicius, 2007; Taymoori and Berry, 2009). However, the findings were not comparable to previous validation studies as mentioned above, as their method of estimation and rotation of the HBM scales were different from the current study. The principal axis factoring was used in this study because it is more robust compared to the principal component analysis (Lawrence and Hancock, 1999). Furthermore, this method attempts to account for error variances (Yong and Pearce, 2013) and much more reliable (Josephine et al., 2015). Since the items were self-developed from the extensive review of other studies that utilized HBM models, it is assumed that the factors extracted are correlated, hence the oblique, Promax rotation method was used (Yong and Pearce, 2013). 

From the original 67 items that underwent factor analysis, a nine-factor solution consisting of 54 items were identified. Cues to action construct was added to the newly developed scale as the construct which is under HBM was not found in the CHBMS questionnaire. The four items of cues to action for BSE (O1, O2, O3 and O4) were deleted during the EFA process because they performed poorly as elements of this instrument. However, this deletion does not indicate that these items were less important as trigger factors but were not statistically relevant to form a factor or subscale in this model. As cues to action is an important trigger for BSE adoption, hence, these items can be further revisited as dichotomized response format (agree and disagree) for the future subscale in HBM (Jirojwong and MacLennan, 2003). The other nine items were deleted due to poor performance on their respective scales as they do not fulfill the criteria of factor loadings of > 0.4 or cross-loaded on multiple factors. 

Nine subscales in the final HBM scale showed good internal consistency with Cronbach alpha > 0.7 indicating the reliability of this scale. This finding is consistent to other studies which reported the Cronbach alpha coefficients of the extracted factors of 0.72 to 0.94 (Lee et al., 2002; Secginli and Nahcivan, 2004; Parsa et al., 2008; Akhtari-zavare et al., 2018). However, the study conducted by Mikail and Petro-Nustas (2001) reflected much lower alpha coefficients with scales ranging from 0.65 to 0.89. The factor loadings in this study were more than 0.4 for all the subscales indicating that all items are integral to the scale and represented the same construct. Furthermore, the correlations between the constructs provided further support in ensuring good construct validity for the 54-item HBM scale which comprised of nine factors that were distinct from each other as their correlation were fair to moderate. 

All subscales showed some evidence of being negatively correlated indicating that there is inverse relationship of the two subscales and positive correlation indicating a synergistic relationship between the subscales. In this study, (between F1 and F2; those who have high self-efficacy tend to have lower barriers in performing mammogram screening) and (between F1 and F5; those who received stronger stimuli had the ability or self-confidence in performing the mammogram). This was expected as participants were asked to respond in performing BSE and mammogram as either favourable or unfavourable. The higher score in any dimension of HBM scale meant that the women were more likely to adopt BSE and or mammogram screening behavior. Using HBM as a foundation, health care providers could then develop targeted support services or reminder alert for women and their families such as medical education or counselling on BSE and mammogram screening. 

Findings from this study revealed adequate estimates of validity and internal consistency reliability for the BSE and screening mammogram using HBM. It is believed that this questionnaire can be utilized by healthcare professionals to assess women’s health beliefs, education and the support needed amongst women in Kuantan, Pahang to participate in early breast cancer screening and detection. As this study is at the exploratory phase, confirmatory factor analysis (CFA) will be carried out randomly on 520 women living in Kuantan, Pahang that fulfills the study criteria using structured equation modelling (SEM). 

Principal axis factoring (PAF) and maximum likelihood factor analysis (MLFA) are the commonest estimation methods utilized in EFA. Principal axis factoring is known to be able to recover weak factors whilst MLFA is equally efficient. As such, no evidence can be obtained regarding which method to choose for types of factor patterns and sample sizes (Winter and Dodou, 2012). The issue of sample size for EFA is not so straightforward because a minimum sample size cannot be obtained analytically as the procedure involves a high degree of subjectivity. To date, most of the sample size calculations for EFA were rules derived from experts’ experience (Pearson and Mundfrom, 2010). The most frequently cited guidelines utilized absolute numbers such as sampling of at least 100 subjects (Gorsuch, 1983; Kline, 1994), scale of sample size adequacy (Comrey and Lee, 1992) and subjects per variable (Gorsuch, 1983; Everitt, 1975; Nunnally, 1978). 

Due to the requirement of the project, questions on health belief on breast cancer, health belief on BSE and health belief on mammography were all grouped into one common questionnaire. As a result of this, the questionnaire appears to be very lengthy. However, individual sections of the questionnaire can be utilized on its own for future studies. As such, the 54-item HBM scale of BSE and mammogram screening can be further utilized in a larger sample of Malaysian women population or culturally diverse population to further explore its psychometric properties. 

The authors wish to express their sincere appreciation to the Clinical Research Centre, HTAA for the help rendered in validating the questionnaire. We further acknowledged the contribution from the postgraduate students, Aaina Mardhiah Abdul Mutalib and Hanis Aisyah Ramli for their help in the construction of the questionnaire and the collection of data for the exploratory study. We also wish to thank all participants for participating in the study. This study is funded by the Fundamental Research Grant (FRGS 17-002-0568), Ministry of Higher Education Malaysia. The study protocol was approved by the IIUM Research Ethics Committee (IREC 2017-075) and Malaysia Research Ethics Committee (NMRR-17-2131-37586 (IIR)). 

## Conflict of Interest Disclosure Statement

This research is sponsored by the Fundamental Research Grant (FRGS 17-002-0568), Ministry of Higher Education Malaysia and we are reporting that we have no financial or business interest in whatsoever company that may be affected by the research reported in the enclosed paper. 

## References

[B1] Akhtari-zavare M, Aliyan-fini F, Ghanbari-baghestan A, Mohd-sidik S (2018). Development and validation of breast cancer knowledge and beliefs questionnaire for Malaysian student population. Pertanika J Soc Sci Hum.

[B2] Befort CA, Nazir N, Engelman K, Choi W (2013). Fatalistic cancer beliefs and information sources among rural and urban adults in the United States. J Cancer Educ.

[B3] Bray F, Ferlay J, Soerjomataram I (2018). Global cancer statistics 2018: GLOBOCAN estimates of incidence and mortality worldwide for 36 cancers in 185 countries. CA Cancer J Clin.

[B4] Champion VL (1984). Instrument development for health belief model constructs. Adv Nurs Sci.

[B5] Chukmaitov A, Wan TTH, Menachemi N, Cashin C (2008). Breast cancer knowledge and attitudes towards mammography as predictors of breast cancer preventive behavior in Kazakh, Korean, and Russian women in Kazakhstan. Int J Public Health.

[B7] Connelly LM (2008). Pilot studies. Medsurg Nurs.

[B8] Everitt B (1975). Multivariate analysis: The need for data, and other problems. Br J Psychiatry.

[B9] Farid NDN, Aziz NA, Al-Sadat N, Jamaludin M, Dahlui M (2014). Clinical breast examination as the recommended breast cancer screening modality in a rural community in Malaysia; What are the factors that could enhance its uptake?. PLoS One.

[B10] Fulton JP, Buechner JS, Scott HD (1991). A study guided by the Health Belief Model of the predictors of breast cancer screening of women ages 40 and older. Public Health Rep.

[B11] George SA (2000). Barriers to breast cancer screening: An integrative review. Health Care Women Int.

[B13] Gullatte MM, Brawley O, Kinney A, Powe B, Mooney K (2010). Religiosity, spirituality and cancer fatalism beliefs on delay in breast cancer diagnosis in African American women. J Relig Health.

[B15] Jirojwong S, MacLennan R (2003). Health beliefs, perceived self-efficacy, and breast self-examination among Thai migrants in Brisbane. J Adv Nurs.

[B16] Josephine NN, Kihoro JM, Anthony W (2015). Principal component and principal axis factoring of factors associated with high population in Urban areas: A case study of Juja and Thika, Kenya. Am J Theoretical Appl Stat.

[B19] Kulakci H, Ayyildiz TK, Yildirim N (2015). Effects of breast cancer fatalism on breast cancer awareness among nursing students in Turkey. Asian Pac J Cancer Prev.

[B20] Lawrence FR, Hancock GR (1999). Conditions affecting integrity of a factor solution under varying degrees of overextraction. Educ Psychol Meas.

[B21] Lee EH, Kim JS, Song MS (2002). Translation and validation of Champion’s Health Belief Model Scale with Korean women. Cancer Nurs.

[B22] Mahmud A, Aljunid SM (2018). Availability and accessibility of subsidized mammogram screening program in peninsular Malaysia: A preliminary study using travel impedance approach. PLoS One.

[B23] Marmara D, Marmara V, Hubbard G (2017). Health beliefs, illness perceptions and determinants of breast screening uptake in Malta: A cross-sectional survey. BMC Public Health.

[B24] Mikail BI, Petro-Nustas WI (2001). Transcultural adaptation of Champion’s Health Belief Model Scales. J Nurs Scholarsh.

[B27] Mirzaei-Alavijeh M, Ghorbani P, Jalilian F (2018). Socio-cognitive determinants of the mammography screening uptake among Iranian women. Asian Pac J Cancer Prev.

[B28] Mohamed HAE, Ibrahim YM, Lamadah SM, El-Magd MHA (2016). Application of the health belief model for breast cancer screening and implementation of breast self-examination educational program for female students of selected medical and non-medical faculties at Umm al Qura University. Life Sci J.

[B30] Noroozi A, Jomand T, Tahmasebi R (2011). Determinants of breast self-examination performance among Iranian women: An application of the health belief model. J Cancer Edu.

[B32] Parsa P, Kandiah M, Mohd Nasir MT, Hejar AR, Nor Afiah MZ (2008). Reliability and validity of Champion’s Health Belief Model Scale for breast cancer screening among Malaysian women. Singapore Med J.

[B33] Pearson RH, Mundfrom DJ (2010). Recommended sample size for conducting exploratory factor analysis on dichotomous data. J Mod Appl Stat Methods.

[B34] Secginli S, Nahcivan NO (2004). Reliability and validity of the breast cancer screening belief scale among Turkish women. Cancer Nurs.

[B35] Shah NM, Lim BTN, Hui NY, Islahudin FH, Hatah EM (2017). Knowledge and perception of breast cancer and its treatment among Malaysian women: Role of religion. Trop J Pharm Res.

[B36] Shahroodi MV, Pourhaje F, Esmaily H, Pourhaje F (2015). The relationship between breast self-examination and stages of change model in health volunteers. J Res Health.

[B37] Stewart SL, Rakowski W, Pasick RJ (2009). Behavioral constructs and mammography in five ethnic groups. Health Educ Behav.

[B40] Taymoori P, Berry T (2009). The validity and reliability of Champion’s Health Belief Model Scale for breast cancer screening behaviors among Iranian women. Cancer Nurs.

[B42] Winter JCF, Dodou D (2012). Factor recovery by principal axis factoring and maximum likelihood factor analysis as a function of factor pattern and sample size. J Appl Stat.

[B43] Yarbrough SS, Braden CJ (2001). Utility of health belief model as a guide for explaining or predicting breast cancer screening behaviours. J Adv Nurs.

[B44] Yong AG, Pearce S (2013). A beginner’s Guide to factor analysis: focusing on exploratory factor analysis. Tutor Quant Methods Psychol.

[B45] Yucel SC, Orgun F, Tokem Y, Avdal EU, Demir M (2014). Determining the factors that affect breast cancer and self-breast examination beliefs of Turkish nurses in academia. Asian Pac J Cancer Prev.

[B46] Yusof A, Chia YC, Hasni YM (2014). Awareness and prevalence of mammography screening and its predictors – A cross sectional study in a primary care clinic in Malaysia. Asian Pac J Cancer Prev.

[B47] Zelviene A, Bogusevicius A (2007). Reliability and validity of the Champion’s Health Belief Model scale among Lithuanian women. Cancer Nurs.

